# Effect of ruminally protected lysine on growth and nitrogen balance of grain-finished beef cattle fed diets of decreasing protein concentration

**DOI:** 10.1093/tas/txag076

**Published:** 2026-06-13

**Authors:** Donald S Herzog, Janghoon Jo, Daiki Nakajima, Kazuki Nakagawa, Rodrigo de Nazare Santos Torres, Lester J Nolasco, Jon P Schoonmaker

**Affiliations:** Department of Animal Science, Purdue University, West Lafayette, IN, United States; Department of Animal Science, Purdue University, West Lafayette, IN, United States; Ajinomoto Inc., Kanagawa, Japan; Ajinomoto Inc., Kanagawa, Japan; Department of Animal Science, Purdue University, West Lafayette, IN, United States; Department of Animal Science, Purdue University, West Lafayette, IN, United States; Department of Animal Science, Purdue University, West Lafayette, IN, United States

**Keywords:** amino acid, beef cattle, feedlot, lysine, nitrogen balance, protein

## Abstract

Two experiments were conducted to determine the effect of ruminally protected lysine (AjiPro-L) supplementation on performance and nitrogen (N) balance of feedlot cattle fed decreasing concentrations of dietary protein. Diets contained (DM basis) 68.3% to 72.2% corn, 10% grass silage, 5.5% supplement, and soybean co-products (meal, hulls). Treatments consisted of a standard protein control diet (14.4% CP, CON), a reduced crude protein (CP) diet supplemented with 0.163% AjiPro-L (12.4% CP, RCP1), and a reduced CP diet supplemented with 0.315% AjiPro-L (11.6% CP, RCP2). In experiment one, 90 steers were allotted by weight (352 ± 13.8 kg) to 1 of 3 treatments to determine the effect of diets on performance and carcass characteristics. Steers were slaughtered at a target BW of 642 kg. In experiment two, 6 heifers (393 ± 10.9 kg) were allotted to the same diets in a replicated 3 × 3 Latin square design (28-d periods) to determine the effect of diets on N balance. Urine and feces were collected and quantified daily during the last 4 days of each period using a urinary catheter and fecal bag. Statistical analyses were conducted using the GLIMMIX procedure of SAS. Decreasing CP and adding lysine to high grain feedlot diets did not affect weight, average daily gain, dry matter intake, gain:feed, or days on feed (*P* ≥ 0.11) and did not affect blood concentrations of total protein, urea N, alanine aminotransferase, or triglyceride (*P* ≥ 0.54). Diet did not affect hot carcass weight, fat depth, longissimus muscle area, KPH%, or marbling score (*P* ≥ 0.22). Steers fed RCP2 had a decreased dressing percentage compared to steers fed CON and RCP1 (*P* = 0.01). Steers fed RCP1 and RCP2 tended produced carcasses with a decreased yield grade (*P* = 0.10) compared with steers fed the CON diet. Heifers fed RCP2 excreted less total N (*P* = 0.04) and tended to excrete less urinary N (*P* = 0.07) compared with heifers fed CON. Total and urinary N excretion did not differ for heifers fed RCP1 compared to heifers fed CON or RCP2 (*P* ≥ 0.10). The percentage of N excreted in feces was greater for heifers fed RCP2 compared with heifers fed CON or RCP1 (*P* = 0.04). In conclusion, decreasing dietary CP to 11.6% in high grain feedlot diets and adding lysine to meet amino acid requirements decreased N excretion, improved carcass leanness, and did not alter animal performance or blood parameters.

## Introduction

Exceeding the Nutrient Requirements of Beef Cattle (NASEM 2016) recommendations for crude protein (CP) is common practice in grain-finishing systems (Samuelson et al. 2016). Overfeeding nitrogen (N) is considered a low-cost, low-risk strategy to safeguard cattle performance and health by accounting for variation in genetic potential within and across pens (Cowley et al. 2019). High-protein corn co-products (eg, distillers grains, corn gluten feed) are frequently included in finishing diets and can substantially increase dietary CP while providing rumen undegradable protein. However, these ingredients may not fully correct imbalances in essential amino acids, particularly lysine, in corn-based systems. Nitrogen utilization in beef cattle is inefficient: only 10% to 20% of ingested N is retained in tissues, 30% to 50% is excreted in feces, and the majority, 40% to 70%, is eliminated in urine (Cole and Todd 2009). This inefficiency contributes to environmental challenges, including N runoff and ammonia emissions (Abbasi et al. 2018).

The most effective approach to decrease N excretion into the environment is to decrease N intake of animals through precision feeding and application of the metabolizable protein system. Indeed, decreasing dietary crude protein content decreases nitrogen excretion and ammonia emissions (Vasconcelos et al. 2009; Batista et al. 2016; Devant et al. 2022). However, this must be done without compromising animal performance as has been reported in some cases (Cooper et al. 2002; Gleghorn et al. 2004). To accomplish this, it is important to provide adequate ruminal N as well as avoid amino acid imbalances in decreased crude protein (CP) diets, as these can lead to and inefficient nitrogen utilization. Inadequate ruminal N can limit microbial protein synthesis, reducing metabolizable protein supply, whereas imbalances in essential amino acids can further constrain the efficient use of absorbed protein. A deficiency in any specific amino acid can limit the use of others, even when they are present in adequate amounts (Cole and Van Lunen 1994; Klemesrud et al. 2000a). Such imbalances or deficiencies decrease a ruminant’s ability to effectively utilize metabolizable protein, ultimately impairing growth performance and overall productivity (Harper et al. 1964) as well as leading to N excretion. In corn-based rations, which are common in feedlot settings, lysine is typically the first limiting amino acid (Klemesrud et al., 2000). Although ruminally protected lysine has shown promise in some beef cattle applications to improve average daily gain (Klemesrud et al. 2000a, 2000b; Xue et al. 2011) and carcass leanness (Teixeira et al. 2019; Cabezas et al. 2023), previous research has not adequately explored using supplemental lysine to target decreased dietary protein resulting in less nitrogen excretion.

This study addresses that gap by investigating whether supplementing a grain-based diet with rumen-protected lysine allows for a reduction in dietary CP content and nitrogen excretion without negatively impacting growth performance or carcass traits. We hypothesized that supplementing rumen protected lysine would maintain performance and enhance nitrogen efficiency in beef cattle fed reduced-CP diets. To test this, we conducted two experiments: one to evaluate the effects of rumen-protected lysine supplementation on growth performance, carcass characteristics, and blood biochemistry in grain-finished steers fed diets with decreasing CP concentrations, and another to assess nitrogen balance in heifers fed the same diets.

## Materials and methods

This study was performed at the Purdue University Animal Sciences Research and Education Center (ASREC) in West Lafayette, IN. All procedures involving animal care and management were approved by the Purdue University Animal Care and Use Committee and were in accordance with the Guide for the Care and Use of Agricultural Animals in Agricultural Research and Teaching (FASS 2010).

### Experiment 1 – performance study

Ninety Angus × Simmental steers, sourced from a sale barn in Kentucky, were allotted by body weight (352 ± 13.8 kg) to 3 treatments to determine the effect of ruminally protected lysine (AjiPro-L Generation 3) supplementation on performance and carcass characteristics of feedlot cattle fed decreasing concentrations of dietary protein. Thirty steers were fed each diet and steers were housed in pens of six (5 pens per treatment). Pens (6.1 × 3.3 m) were located in a curtain-sided, slatted-floor finishing barn, and provided 30 cm of bunk space per animal. Treatments consisted of a standard protein control diet (14.4% CP, CON), a reduced crude protein (CP) diet supplemented with 0.163% AjiPro-L (12.4% CP, RCP1), and a reduced CP diet supplemented with 0.315% AjiPro-L (11.6% CP, RCP2). A reduced CP diet without lysine supplementation was not evaluated. Diets (Table 1) were formulated on a DM basis to meet or exceed NASEM (2016) vitamin and mineral requirements of 500 kg steers predicted to gain 1.7 kg/d. Diets contained (DM basis) 68.3% to 72.2% corn, 10% grass silage, 5.5% supplement, and soybean co-products (meal, hulls). The rumen-protected lysine product (AjiPro-L Generation 3) contained 42% L-lysine-HCl with 80% ruminal escape and 80% intestinal digestibility (Fehlberg et al. 2020).

**Table 1:** Diet composition (DM basis).^a^

	Control	RCP1	RCP2
***Corn***	68.3	70.0	71.2
***Grass Silage***	10.0	10.0	10.0
***Soybean Hulls***	6.7	9.0	10.0
***Soybean Meal***	9.5	4.5	2.3
***Vitamin/mineral Supplement^b^***	5.5	5.5	5.5
***AjiPro-L Supplement^c^***	0.0	1.0	1.0
***Nutrient composition^d^***			
*** Crude protein***	14.4	12.4	11.6
*** RDP^e^, % of crude protein***	61.4	59.9	59.0
*** RUP^e^, % of crude protein***	38.6	40.1	41.0
*** Lysine supply, g/d***	62.7	62.9	62.8
*** Lysine required, g/d***	46.5	46.7	46.9
*** NDF, %***	19.29	20.46	20.96
*** NEm, Mcal/kg^f^***	2.05	2.05	2.05
*** NEg, Mcal/kg^f^***	1.39	1.39	1.39
*** Ca, %***	0.64	0.64	0.63
*** P, %***	0.35	0.33	0.32

a Treatments were (1) a diet with no lysine supplementation (Control), (2) rumen-protected lysine (25% metabolizable lysine, AjiPro-L, Ajinomoto Heartland) included at 0.163% (RCP1), and (3) rumen-protected lysine (AjiPro-L) included at 0.315% (RCP2).

b Vitamin/mineral supplement contained (DM basis): 9.1% urea, 7.54% Ca, 1.45% K, 0.65% P, 0.29% Mg, 0.20% S, 745.28 ppm Zn, 258.65 ppm Fe, 531.89 ppm Mn, 157.94 ppm Cu, 14.15 ppm I, 4.18 ppm Se, 1.73 ppm Co, 12.05 IU/g vitamin A, 1.2 IU/g vitamin D, 0.03 IU/g vitamin E, 87.7 ppm Monensin (Rumensin, Elanco Animal Health, Indianapolis, IN).

c The RCP1 supplement contained 85.37% ground corn and 14.63% AjiPro-L; The RCP2 supplement contained 71.19% ground corn and 28.81% AjiPro-L.

d Analyzed at Cumberland Valley Analytical Services (Waynesboro, PA).

e RDP, Rumen degradable protein; RUP, Rumen undegradable protein.

f Calculated based on NASEM (2016).

Steers were vaccinated and given a booster against Infectious Bovine Rhinotracheitis, Bovine Viral Diarrhea Types I and II, Parainfluenza-3, Bovine Respiratory Syncytial Virus, *Mannheimia haemolytica*, and *Pasturella multocida* (Vista Once, Merck Animal Health, Summit, NJ), against *Clostridia* and *Haemophilus somnus* (Vision-7 Somnus; Merck Animal Health), treated with a pour-on (Cydectin, Bayer, Shawnee Mission, KS) for external parasites, and drenched with a de-wormer (Safeguard, Merck Animal Health, Madison, NJ) for internal parasites. Cattle were implanted with Revalor-XS (20 mg trenbolone acetate and 4 mg estradiol, Merck Animal Health, Madison, NJ) at feedlot entry. Steers were weighed twice on consecutive days at the initiation of the study (day 0) and at slaughter and weighed once approximately every 28 days to monitor growth and health. Scales (480 Legend, Rice Lake Weighing Systems, Rice Lake, WI) weighed to the nearest 0.5 kg and were checked for accuracy at each weigh date. Feed intake was recorded daily and average daily gain, dry matter intake, and feed efficiency were calculated for day 0 to 84, day 85 to slaughter, and for day 0 to slaughter. Feed was delivered once daily at 0900 h, and steers were allowed *ad libitum* access to feed and water. Feed was delivered using a tractor and 4 auger feed mixer wagon (Kuhn Knight 4136 Botec; Kuhn Inc., Brodhead, WI) equipped with a digital scale indicator (Digi-Star, Ft. Atkinson, WI) located in the cab of the tractor that was connected to load cells in the feed-mixer wagon. Mixer wagon scales weighed to the nearest 0.91 kg. Daily feed deliveries were adjusted using a 4-point bunk scoring system (Pritchard 1993) to allow for *ad libitum* feed intake with little or no accumulation of unconsumed feed (score of ≤ 1). Feed delivery was recorded daily, and diet ingredient samples were collected every two weeks for DM and nutrient analysis. Any feed refusals were weighed back and subtracted from pen intakes. As-fed formulations were adjusted for DM content accordingly every other week. Subsamples of twice monthly diet ingredients were composited and analyzed by wet chemistry for CP, NDF, P, and Ca (Cumberland Valley Analytical Services, Waynesboro, PA). Feed intake, ADG, and gain:feed were determined for days 0 to 84, 85 to slaughter, and overall from day 0 to slaughter.

On days 0, 28, 56, 84, 112, and at slaughter, blood was collected via jugular venipuncture into serum tubes (BD Vacutainer; Becton Dickinson, Franklin Lakes, NJ), allowed to clot for 30 to 45 minutes at room temperature, and centrifuged at 1250 × g for 15 min. Serum was separated and stored at −20°C until analysis. Serum was analyzed using commercially available bovine specific assay kits, read with a Tecan Spark 10M multimode microplate reader (Tecan Trading AG, Mannedorf, Switzerland). Serum urea nitrogen (SUN) was read at 530 nm (diacetylmonoxime kit, Stanbio Laboratory, Boerne, TX). Total protein was read at 562 nm (Pierce BCA kit, Thermo Fisher, Waltham, MA), albumin was read at 430 nm (ELISA kit, Biomatik, Kitchener, ON, Canada), and globulin was determined as the difference between total protein and albumin. Alanine amino transferase (colorimetric activity assay kit, Cayman, Ann Arbor, MI) and aspartate amino transferase (colorimetric activity assay kit, Cayman) were read at 340 nm. Triglyceride was read at 540 nm (colorimetric activity assay kit, Cayman) and cholesterol was read at an excitation wavelength between 530 and 540 nm and emission wavelength between 585-595 nm (fluorometric activity assay kit, Cayman). Intra-assay CV was 0.95, 1.46, 2.04, 2.85, 4.72, 1.28, and 3.52% and inter-assay CV was 1.99, 1.00, 3.66, 0.89, 1.75, 1.36, and 5.48% for SUN, total protein, albumin, ALT, AST, triglyceride, and cholesterol, respectively. Serum amino acids were analyzed using the post-column colorimetric derivatization with the ninhydrin method using a High Speed Amino Acid Analyzer (L-8900; Hitachi High-Tech Corp., Tokyo, Japan).

Cattle were transported 400 km to a commercial abattoir (Tyson Foods Inc., Joslin, IL) for slaughter. Pens of steers were slaughtered at 3 different time points (138, 159 and 194 d) when an average pen body weight of between 591.4 and 661.2 kg was achieved. An equal number of treatment pens were slaughtered at each time point (2, 2, and 1 pen per treatment on days 138, 159, and 194, respectively) and all steers within a pen were slaughtered on the same day. The weighted average of days on feed (pen days) was 154 for steers on all treatments. Hot carcass weight, rib-eye area, fat thickness, yield grade, marbling score, and quality grade were determined 24 h after slaughter by qualified personnel.

Data were analyzed as a complete randomized design using the GLIMMIX procedure of SAS, with pen as the experimental unit. Periodical performance and serum data were analyzed as repeated measures over time by comparing five covariance structures for each variable (variance components, compound symmetric, heterogenous compound symmetric, autoregressive 1, and unstructured). Autoregressive 1 consistently yielded the lowest Bayesian Information Criteria and was used for all results. The repeated measures model included the random effect of pen and the fixed effect of treatment, time, and the interaction of treatment and time. The Satterthwaite approach was used to estimate denominator degrees of freedom. The SLICEDIFF function of SAS was used to analyze only the within time comparisons that were meaningful and the SLICE function of SAS was used to determine the simple effects of treatments within time, which is what is presented in the results section. The least square means statement was used to calculate adjusted means and pairwise treatment comparisons were corrected using the Tukey adjustment. Significance was declared at *P* ≤ 0.05 and a tendency was declared 0.05 < *P* ≤ 0.10.

### Experiment 2 – nitrogen balance

Six heifers (393 ± 10.9 kg) were allotted to the same diets as experiment 1 in a replicated 3 × 3 Latin square design (28-d periods) to determine the effect of diets on N balance. The Latin square was balanced for carry over effects, such that each experimental diet followed a different diet for each respective treatment. Heifers were housed individually in 3.0 × 9.1 m pens inside a 3-sided barn with concrete floors covered with wood shavings. Feed was offered once daily at 0800 h and heifers were allowed ad libitum access to feed and water. Daily feed delivery, DM adjustment, bunk scoring, and nutrient composition analyses were as described for experiment 1.

Heifers were tied to the feed bunk using a halter and urine and feces were collected and quantified daily during the last 4 days of each 28 day period. Feed and water were offered for ad libitum intake throughout the collection period. Urine was collected with a latex Foley catheter (Medline, Northfield, IL), inserted into the bladder, and attached to a 4 L drainage bag (Medline, Northfield, IL), fixed to the stall. Drainage bags were emptied at 3 to 6 h intervals and stored at 4°C in sealed containers. Total urinary output was determined and a 15 mL subsample was collected and stored at −20°C for N analysis for every 24 h period. Feces were collected in waterproof canvas bags connected to the heifer by a leather harness, removed from fecal bags twice daily, and weighed at 0700 h the following morning. A 5% subsample of the total daily fecal weight was collected daily for N analysis. Fecal samples were oven dried at 60°C and ground using a Wiley mill (1-mm screen, Arthur H. Thomas, Philadelphia, PA). Ground feces and urine were analyzed for nitrogen by combustion (Leco TruMac, LECO Corporation, St. Joseph, MI).

Data were analyzed as a replicated 3 × 3 Latin square design using the GLIMMIX procedure of SAS (Version 9.3, SAS Inst. Inc., Cary, NC) with heifer within period considered the experimental unit. Fecal and urinary nitrogen data were analyzed as repeated measures over time by comparing five covariance structures for each variable (variance components, compound symmetric, heterogenous compound symmetric, autoregressive 1, and unstructured). Autoregressive 1 consistently yielded the lowest Bayesian Information Criteria and was used for all results. The repeated measures model included the random effect of heifer and the fixed effect of diet, period, and the interaction between diet and period. The Satterthwaite approach was used to estimate denominator degrees of freedom. The least square means statement was used to calculate adjusted means and pairwise treatment comparisons were corrected using the Tukey adjustment. Significance was declared at *P* ≤ 0.05 and a tendency was declared 0.05 < *P* ≤ 0.10.

## Results

Feedlot performance data is presented in Table 2. Four steers in experiment 1 (1 CON, 2 RCP1, and 1 RCP2) were removed from the study for reasons unrelated to experimental treatments. Steer BW did not differ among treatments at any point during the study (*P* ≥ 0.88). Average daily gain (*P* ≥ 0.43), dry matter intake (*P* ≥ 0.11), and gain:feed (*P* ≥ 0.79) did not differ during the first half of the study (day 0 to 84), the second half of the study (day 85 to slaughter), or overall (day 0 to slaughter).

**Table 2:** Effect of lysine supplementation on performance of feedlot cattle.^a^

	Con	RCP1	RCP2	SEM	*P*-value
***Weight, kg***					
*** Day 0***	353.2	351.3	349.8	12.45	0.98
*** Day 84***	523.6	519.4	514.6	12.45	0.88
*** Slaughter***	642.8	636.4	645.1	12.45	0.88
***ADG, kg***					
*** Day 0 to 84***	2.04	2.02	1.98	0.087	0.87
*** Day 85 to slaughter***	1.66	1.68	1.81	0.087	0.43
*** Day 0 to slaughter***	1.87	1.86	1.90	0.065	0.88
***DMI, kg***					
*** Day 0 to 84***	11.5	11.6	11.0	0.40	0.56
*** Day 85 to slaughter***	12.1	12.3	13.3	0.40	0.11
*** Day 0 to slaughter***	11.7	11.9	12.0	0.36	0.84
***Gain:feed, kg/kg***					
*** Day 0 to 84***	0.179	0.174	0.182	0.0076	0.79
*** Day 85 to slaughter***	0.138	0.137	0.137	0.0076	0.99
*** Day 0 to slaughter***	0.161	0.156	0.158	0.0049	0.84
***Days on feed***	154.0	153.9	154.0	6.93	0.99

a Treatments were (1) a diet with no lysine supplementation (Control), (2) rumen-protected lysine (25% metabolizable lysine, AjiPro-L, Ajinomoto Heartland) included at 0.163% (RCP1), and (3) rumen-protected lysine (AjiPro-L) included at 0.315% (RCP2).

Serum metabolite concentrations are presented in Table 3 as overall means. There were no differences in serum concentrations of any of the metabolites (*P* ≥ 0.28). Serum amino acid concentrations are presented in Table 4 as overall means. Branched chain amino acids differed or tended to differ. Steers fed RCP1 had greater serum valine (*P* = 0.03) concentrations and tended to have greater serum leucine and isoleucine (*P* ≤ 0.10) concentrations compared to steers fed RCP2. Serum concentrations of valine, leucine, and isoleucine were intermediate for steers fed the control diet did not differ from steers fed RCP1 or RCP2 (*P* > 0.10). Concentrations of other amino acids as well as total, essential, and non-essential amino acids did not differ among treatments (*P* ≥ 0.14).

**Table 3:** Effect of lysine supplementation on blood biochemistry (data from dates combined).^a^

	Con	RCP1	RCP2	SEM	*P*-value
***Total protein, mg/mL***	86.6	89.3	89.1	2.59	0.69
***Albumin, mg/mL***	31.7	34.5	32.0	2.60	0.71
***Globulin, mg/mL***	54.7	55.3	56.8	2.81	0.85
***Albumin:Globulin***	0.79	0.92	0.77	0.155	0.76
***Blood urea nitrogen, mg/dL***	17.6	16.4	17.9	1.68	0.79
***Alanine aminotransferase, U/L***	27.6	26.9	28.3	1.61	0.83
***Aspartate aminotransferase, U/L***	31.9	34.2	34.6	1.23	0.28
***Triglyceride, mg/dL***	17.3	17.3	15.8	1.10	0.54
***Cholesterol, g/dL***	109.0	119.5	113.0	6.23	0.50

a Treatments were (1) a diet with no lysine supplementation (Control), (2) rumen-protected lysine (25% metabolizable lysine, AjiPro-L, Ajinomoto Heartland) included at 0.163% (RCP1), and (3) rumen-protected lysine (AjiPro-L) included at 0.315% (RCP2).

**Table 4:** Effect of lysine supplementation on blood amino acids.^a^

*Amino acids, mg/mL*	Con	RCP1	RCP2	SEM	*P*-value
***Alanine***	31.5	32.3	30.8	1.76	0.82
***Arginine***	16.8	15.7	17.0	0.56	0.25
***Asparagine***	3.34	3.19	3.13	0.125	0.49
***Aspartate***	0.41	0.47	0.38	0.038	0.28
***Citrulline***	7.60	7.62	7.36	0.299	0.79
***Cysteine***	0.013	0.014	0.016	0.0020	0.53
***Glutamate***	10.4	11.4	9.8	0.58	0.21
***Glutamine***	31.7	31.6	29.9	0.88	0.29
***Glycine***	31.5	31.7	32.8	1.30	0.75
***Histidine***	7.09	7.45	6.97	0.286	0.49
***3-Methyl-Histidine***	0.71	0.71	0.77	0.043	0.49
***Isoleucine***	10.8^bc^	11.3^b^	10.1^c^	0.34	0.10
***Leucine***	18.5^bc^	20.8^b^	18.3^c^	0.81	0.09
***Lysine***	11.5	12.1	11.3	0.57	0.60
***Methionine***	2.77	3.03	2.69	0.116	0.14
***Ornithine***	8.20	8.81	7.77	0.371	0.18
***Phenylalanine***	6.69	7.48	7.07	0.272	0.15
***Proline***	9.53	9.94	8.98	0.483	0.39
***Serine***	10.2	10.3	10.1	0.44	0.93
***Taurine***	7.26	8.36	8.03	0.649	0.49
***Threonine***	6.84	7.46	6.61	0.315	0.18
***Tryptophan***	4.35	4.81	4.47	0.158	0.14
***Tyrosine***	6.52	7.06	6.51	0.237	0.21
***Valine***	25.7^bc^	26.8^b^	23.1^c^	0.87	0.03
***Essential***	110.9	116.8	107.5	3.11	0.14
***Non-Essential***	158.9	163.4	156.6	4.42	0.55
***Total***	269.8	280.2	263.7	7.18	0.29

a Treatments were (1) a diet with no lysine supplementation (Control), (2) rumen-protected lysine (25% metabolizable lysine, AjiPro-L, Ajinomoto Heartland) included at 0.163% (RCP1), and (3) rumen-protected lysine (AjiPro-L) included at 0.315% (RCP2).

b,c Values within a row without common superscripts differ at the indicated *p*-value.

Carcass characteristics are presented in Table 5. Hot carcass weight, fat depth, rib-eye area, KPH%, and marbling score did not differ among treatments (*P* ≥ 0.22). Steers fed RCP2 produced carcasses with a decreased dressing percentage compared to steers fed Con and RCP1 (*P* = 0.01). Steers fed RCP1 and RCP2 produced carcasses that tended to have decreased yield grades compared to steers fed Con (P = 0.10).

**Table 5:** Effect of lysine supplementation on carcass characteristics of feedlot cattle.^a^

	Con	RCP1	RCP2	SEM	*P*-value
***Hot carcass weight, kg***	399.9	394.2	393.8	5.41	0.68
***Dressing, %***	62.4^b^	62.1^b^	61.3^c^	0.24	0.01
***Fat depth, cm***	1.57	1.39	1.43	0.076	0.22
***Rib-eye area, cm^2^***	86.9	88.7	88.5	1.07	0.45
***Kidney, pelvic, heart fat, %***	1.9	1.9	1.9	0.04	0.99
***Yield grade***	3.5^a^	3.1^c^	3.2^c^	0.11	0.10
***Marbling score***	376.4	383.1	375.3	13.67	0.91

a Treatments were (1) a diet with no lysine supplementation (Control), (2) rumen-protected lysine (25% metabolizable lysine, AjiPro-L, Ajinomoto Heartland) included at 0.163% (RCP1), and (3) rumen-protected lysine (AjiPro-L) included at 0.315% (RCP2).

b,c Values within a row without common superscripts differ at the indicated *p*-value.

Nitrogen balance data are presented in Table 6. Nitrogen intake differed among all 3 treatments (*P* = 0.0001); heifers fed RCP2 had the least N intake, followed by heifers fed RCP1, and heifers fed Con had the greatest N intake. Heifers fed RCP2 decreased total nitrogen excretion (g/d) compared to Con and RCP1 heifers (*P* = 0.04), but heifers fed RCP1 did not decrease total nitrogen excretion compared to heifers fed Con (*P* ≥ 0.10). The decrease in total nitrogen excretion (g/d) caused by feeding RCP2 was due to a decrease in urinary N excretion. Heifers fed RCP2 tended to decrease urine nitrogen excretion (g/d) compared to Con and RCP1 (*P* = 0.09), while heifers fed RCP1 did not decrease urine nitrogen excretion compared to heifers fed Con (*P* ≥ 0.10). Fecal N excretion did not differ among treatments (*P* ≥ 0.20). The percentage of N excreted in feces decreased in heifers fed RCP1 compared with heifers fed Con or RCP2 (*P* = 0.04).

**Table 6:** Effect of lysine supplementation on nitrogen balance.^a^

	Con	RCP1	RCP2	SEM	*P*-value
**N *intake, g/d***	195.6^b^	171.4^c^	154.2^d^	1.56	0.0001
**N *excretion, g/d***	135.7^b^	128.2^ac^	112.3^c^	7.88	0.04
***Fecal excretion***					
*** DM, kg/d***	2.22	2.21	2.36	0.204	0.74
*** Total* N*, g/d***	57.3	47.3	55.0	3.98	0.20
*** Total* N*, %* N *excretion***	42.5^b^	39.4^b^	47.0^c^	2.06	0.04
***Urinary excretion***					
*** Volume, L/d***	39.6	38.2	32.4	9.32	0.73
*** Total* N*, g/d***	79.1^b^	77.5^bc^	63.5^c^	6.73	0.09
*** Total* N*, %* N *excretion***	58.0	58.2	57.3	4.00	0.98
***Retained* N*, g/d***	63.1^b^	45.9^c^	45.1^c^	7.16	0.06

a Treatments were (1) a diet with no lysine supplementation (Control), (2) rumen-protected lysine (25% metabolizable lysine, AjiPro-L, Ajinomoto Heartland) included at 0.163% (RCP1), and (3) rumen-protected lysine (AjiPro-L) included at 0.315% (RCP2).

b,c ^,d^Values within a row without common superscripts differ at the indicated *p*-value.

## Discussion

Decreased protein diets have the potential to decrease the environmental impact of beef production, but decreasing the protein content of the diet carries the risk of limiting essential amino acids, which may restrict growth and carcass leanness (Harper et al. 1964). In diets with large proportions of corn, lysine is the first limiting amino acid for growing beef cattle because corn is limited in lysine (Klemesrud et al. 2000b). Thus, we hypothesized that addition of rumen protected lysine to the diet would maintain cattle performance when reduced protein diets were fed. Decreasing protein content to 11.6% (RCP2) in the current study did lessen urinary and thus total nitrogen excretion which is to be expected and is in line with previous studies (Batista et al. 2016; Devant et al. 2022; da Silva et al. 2025). Kamiya et al. (2021) reported that adding rumen protected lysine to diets in which CP was decreased from 15.1% to to 11.7% also decreased N intake, urinary N excretion and total N excretion. In situations where intake is decreased and requirements are more difficult to meet, rumen-protected lysine can serve as a practical safeguard against deficiencies. Indeed, based on NASEM (2016) analyses of diets during the first 28 days of the study, MP (844, 863, and 745 g/d) and lysine (45.1, 50.2, 46.9 g/d) supplied by CON, RCP1, and RCP2 diets did not meet MP (859, 907 and 807 g/d) or lysine (55.1, 58.1, 51.6 g/d) requirements. It is possible that supplementation with rumen-protected lysine compensated for insufficient MP supply in RCP1 and RCP2 diets allowing for dry matter intake and average daily gain to not differ compared to steers fed the CON diet. Metabolizable protein (1057, 1005, and 1024 g/d) and lysine (56.1, 58.3, 64.3 g/d) supplied by CON, RCP1, and RCP2 diets during the remainder of the study exceeded MP (822, 816, and 835 g/d) and lysine (52.6, 52.2, and 53.5 g/d) requirements. Previous studies show that a true MP deficiency decreases intake as well as gain and that cattle respond quadratically to increasing MP, with ADG and gain:feed improving until requirements are met and then plateauing (Cooper et al. 2002; Gleghorn et al. 2004; Sitorski et al. 2019). Adding incremental amounts rumen protected lysine to high grain feedlot diets that were marginally deficient in protein increased average daily gain (Klemesrud et al. 2000a,2000b; Xue et al. 2011). Hussein and Berger (1995) reported that in cattle fed diets containing soybean meal, which is a good source of lysine, supplemental lysine did not improve performance. Recent work demonstrates that supplementing rumen-protected lysine (often with methionine) allows crude protein to be decreased to just above adequacy without compromising intake, gain, or carcass traits, while improving nitrogen use efficiency (Teixeira et al. 2019; Kamiya et al. 2021; Cabezas et al. 2023). Heiderscheit and Hansen (2020) observed that rumen protected lysine supplementation to steers fed excess crude protein diets (13.5%) decreased overall ADG and the first 56 days gain:feed.

Fat thickness, ribeye area, and yield grade are typically unaffected by wide ranges of MP intake spanning from deficiency to adequacy (Gleghorn et al. 2004; Vasconcelos et al. 2009). In the present study, we demonstrated that feeding RCP1 and RCP2 diets tended to decrease yield grade compared to CON diets. Previous work has shown that amino acid supplementation under low crude protein conditions can influence carcass composition and nitrogen utilization. For example, Cabezas et al. (2023) reported increased carcass leanness and muscle protein with supplemental lysine and methionine in cattle fed 10.5% CP diets, and Teixeira et al. (2019) observed decreased yield grade alongside increased longissimus muscle area and reduced fat thickness with lysine supplementation in steers fed 12.5% CP diets. Similarly, increasing lysine supply with abomasal infusion has been associated with greater nitrogen retention in growing cattle fed corn-based diets (Burris et al. 1976; Batista et al. 2016), and improvements in nitrogen retention have also been reported in lambs supplemented with rumen-protected lysine (Oke et al. 1986). However, responses in carcass characteristics to lysine supplementation have been inconsistent, with several studies reporting no effect on fat thickness, ribeye area, or yield grade in cattle fed 11.7% to 13.5% crude protein diets (Klemesrud et al. 2000b; Heiderscheit and Hansen 2020; Kamiya et al. 2021), and no effect when soybean meal is included (Hussein and Berger 1995). In the present study, the decrease in dressing percentage observed for steers fed the most reduced CP diet (RCP2) may reflect a shift in protein deposition toward visceral tissues, which have high rates of protein turnover. This response likely reflects the combined effects of reduced dietary CP and amino acid supplementation.

Concentrations of the branched-chain amino acids (BCAA; leucine, isoleucine, and valine) in blood decreased in the reduced CP diets, which may reflect greater uptake of these amino acids into muscle. Because BCAA are primarily catabolized in muscle rather than the liver (An et al. 2024), decreased circulating concentrations are consistent with increased utilization for protein synthesis in lean tissue. Leucine in particular is a key regulator of mTOR signaling and muscle protein synthesis (An et al. 2024), and similar shifts in BCAA profiles have been reported when lysine was supplemented to feedlot cattle (Teixeira et al. 2019; Heiderscheit and Hansen 2020; Kamiya et al. 2021). Decreased circulating BCAA may also suggest that they could be next limiting amino acids, as is frequently the case for BCAA in non-ruminants fed corn-soy diets after lysine needs are met (Wiltafsky et al. 2010; Ospina-Rojas et al. 2012).

Blood urea nitrogen (BUN) is closely associated with nitrogen excretion (Kohn et al. 2005), such that reductions in excretion are typically accompanied by decreased BUN. In the present study, cattle fed the RCP2 diet excreted less nitrogen, so a corresponding decrease in serum urea nitrogen (SUN) was expected. However, SUN did not differ among treatments, likely because total nitrogen intake (g/d) was not substantially decreased. Indeed, steers fed RCP1 and RCP2 consumed slightly more feed than controls, although differences were not statistically significant. The fact that decreasing dietary protein and supplementing lysine did not affect other serum markers of protein adequacy (total protein, albumin, globulin), liver metabolic stress (ALT, AST), or lipid metabolites (triglycerides, cholesterol), suggests that feedlot cattle can maintain systemic protein status, hepatic function, and energy metabolism under moderately reduced dietary protein concentrations.

In conclusion, decreasing dietary CP decreased urinary and total N excretion in feedlot cattle without compromising growth, consistent with improved N-use efficiency. Supplementation with rumen-protected lysine did not affect performance but did tend to increase carcass leanness when CP was decreased. The decreased dressing percentage observed at the most limited dietary CP concentration may reflect a shift in protein deposition toward visceral tissues. The decrease in circulating BCAA with lysine supplementation suggests greater uptake into muscle and highlights the potential for BCAA to become limiting as amino acid balance is improved in low CP diets. Blood metabolites and liver enzyme activities were unaffected, indicating that protein status and metabolic health were preserved when in moderately reduced CP diets. Collectively, these findings suggest that balancing amino acid supply in reduced-CP diets can safeguard against protein deficiency while improving nitrogen utilization, with potential benefits for the environmental sustainability of grain-finished beef production.

## List of abbreviations

ADG: average daily gain
BW: body weight
BUN: Blood Urea Nitrogen
CP: crude protein
DM: dry matter
DMI: dry matter intake
MP: Metabolizable Protein
N: Nitrogen
NDF: neutral detergent fiber
NEg: net energy for gain
NEm: net energy for maintenance
RDP: Rumen Degradable Protein
RUP: Rumen Undegradable Protein
SUN: Serum Urea Nitrogen
